# Constraints of using historical data for modelling the spatial distribution of helminth parasites in ruminants

**DOI:** 10.1051/parasite/2021042

**Published:** 2021-05-27

**Authors:** Alizée Hendrickx, Cedric Marsboom, Laura Rinaldi, Hannah Rose Vineer, Maria Elena Morgoglione, Smaragda Sotiraki, Giuseppe Cringoli, Edwin Claerebout, Guy Hendrickx

**Affiliations:** 1 Department of Research and Development, Avia-GIS NV 2980 Zoersel Belgium; 2 CREMOPAR, Department of Veterinary Medicine and Animal Production, University of Naples Federico II 80138 Naples Italy; 3 Department of Infection Biology and Microbiomes, Institute of Infection, Veterinary and Ecological Sciences, University of Liverpool L69 7ZX Liverpool United Kingdom; 4 Parasitology Laboratory, Veterinary Research Institute, Hellenic Agricultural Organization DEMETER 57001 Thessaloniki Greece; 5 Laboratory for Parasitology, Faculty of Veterinary Medicine, Ghent University 9820 Merelbeke Belgium

**Keywords:** *Dicrocoelium dendriticum*, Ruminants, Italy, Distribution, Prevalence, Spatial modeling

## Abstract

*Dicrocoelium dendriticum* is a trematode that infects ruminant livestock and requires two different intermediate hosts to complete its lifecycle. Modelling the spatial distribution of this parasite can help to improve its management in higher risk regions. The aim of this research was to assess the constraints of using historical data sets when modelling the spatial distribution of helminth parasites in ruminants. A parasitological data set provided by CREMOPAR (Napoli, Italy) and covering most of Italy was used in this paper. A baseline model (Random Forest, VECMAP^®^) using the entire data set was first used to determine the minimal number of data points needed to build a stable model. Then, annual distribution models were computed and compared with the baseline model. The best prediction rate and statistical output were obtained for 2012 and the worst for 2016, even though the sample size of the former was significantly smaller than the latter. We discuss how this may be explained by the fact that in 2012, the samples were more evenly geographically distributed, whilst in 2016 most of the data were strongly clustered. It is concluded that the spatial distribution of the input data appears to be more important than the actual sample size when computing species distribution models. This is often a major issue when using historical data to develop spatial models. Such data sets often include sampling biases and large geographical gaps. If this bias is not corrected, the spatial distribution model outputs may display the sampling effort rather than the real species distribution.

## Abstract (fr)

*Dicrocoelium dendriticum* est un trématode qui infecte les ruminants et nécessite deux hôtes intermédiaires différents pour terminer son cycle de vie. La modélisation de la distribution spatiale de ce parasite peut aider à améliorer sa gestion dans les régions à haut risque. L’objectif de cette recherche était d’évaluer les contraintes liées à l’utilisation d’ensembles de données historiques lors de la modélisation de la distribution spatiale des helminthes parasites chez les ruminants. Un ensemble de données parasitologiques fourni par CREMOPAR (Naples, Italie) et couvrant la majeure partie de l’Italie a été utilisé dans cet article. Un modèle de base (Random Forest, VECMAP^®^) utilisant l’ensemble des données a d’abord été utilisé pour déterminer le nombre minimal de points de données nécessaires pour construire un modèle stable. Ensuite, des modèles de distribution annuelle ont été calculés et comparés au modèle de référence. Le meilleur taux de prédiction et le meilleur résultat statistique ont été obtenus pour 2012 et le plus mauvais pour 2016, malgré le fait que la taille de l’échantillon du premier était nettement plus petite que celle du second. Nous discutons comment cela peut s’expliquer par le fait qu’en 2012, les échantillons étaient plus uniformément répartis géographiquement, alors qu’en 2016, la plupart des données étaient fortement regroupées. On conclut que la distribution spatiale des données d’entrée semble être plus importante que la taille réelle de l’échantillon lors du calcul des modèles de distribution des espèces. C’est souvent un problème majeur pour développer des modèles spatiaux quand on utilise des données historiques. Ces ensembles de données comportent souvent des biais d’échantillonnage et de grandes lacunes géographiques. Si ce biais n’est pas corrigé, les résultats du modèle de distribution spatiale peuvent représenter l’effort d’échantillonnage plutôt que la distribution réelle des espèces.

## Introduction

The lancet liver fluke *Dicrocoelium dendriticum* is a parasite of the bile ducts and gallbladder of different mammalian species (mainly ruminants), including humans [31, 34].

The life cycle of this parasite requires two invertebrate intermediate hosts: one being a xerophilic terrestrial snail (of various genera such as *Helicella*, *Zebrina* or *Cernuella*), and the other an ant (mainly of the genus *Formica*) [25].

Clinical signs in ruminants are not usually manifest, even in severe infections, and therefore, major lesions, due to liver impairment are detectable only at post-mortem examination [31, 34]. Lesions are directly proportional to the parasitic burden [23] and chronic inflammation of the bile ducts [8]. In the early stages of the infection, reduced weight gain can be detected, but the infection is usually asymptomatic [34] resulting only in livers being discarded during meat inspection at slaughterhouses or with an appropriate coprodiagnostic analysis [35]. In severe cases, infection can lead to emaciation, anaemia with economic losses in production, and viscera condemnation in animals [2, 14, 35]. Therefore, *D. dendriticum* is, together with *Fasciola hepatica*, one of the leading causes of discarded livers in the abattoir, with associated economic losses. Dicrocoeliosis is also zoonotic [22, 36]. Hence, modelling the spatial distribution of this parasite can help to improve its management in higher risk regions [11, 27]. Due to global climate change, seasonal and spatial patterns of parasites can alter [7]. This also includes indirect effects of climate change such as management changes [32]. Extended grazing periods, animal movements and anti-helminthic resistance leading to treatment failure are important drivers that boost the presence of parasites [5, 15, 19, 28].

Development of *D. dendriticum* is distinctly dependent on ecology, geo-climatic factors and anthropogenic factors. This is mainly due to its intermediate hosts that require highly specific environmental niches such as calcareous or alkaline soils [29]. This results in a widespread presence of this trematode throughout Europe with locally heterogeneous spatial distribution patterns and a significant variation in local prevalence [25].

The environment affects the phases of the parasite lifecycle. Therefore, it is important to include these factors while making risk maps. The environmental factors are geolocated [18]. Species distribution modelling (SDM), also known as environmental modelling, is a tool that combines different observations of species presence or absence with environmental predictors such as temperature, rainfall, elevation, soil type, and vegetation that are ecologically-relevant to the species being modelled [12].

**Figure 1:**
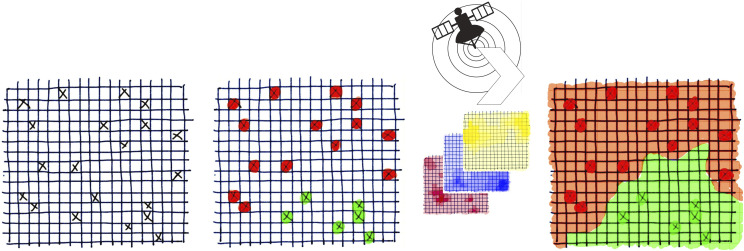
General approach to designing species distribution maps.

The general approach to designing species distribution maps is shown in Figure 1. First, a number of grid-cells (A), in this case farms, are randomly selected within a larger area and are sampled to obtain occurrence data (presence = red, absence = green) (B). Second, a set of environmental data provides information for all the pixels in the sample area (C). These are termed co-variates or predictor variables. Finally, different modelling methods can be used to predict the probability of occurrence of trematodes within each of the grid-cells, which generates a risk map covering the entire area (D).

To develop high-accuracy risk maps, it is pivotal to use the right combination of predictors [13]. Clustering can be observed for *D. dendriticum* in the southern part of Italy mainly due to the specific environmental needs of the intermediate hosts [6, 29]. Ekstam et al. [11] and Musella et al. [29] showed that the prevalence of *D. dendriticum* increases in areas with woody vegetation and decreases in wet areas. Species distribution model algorithms and the accuracy of model output are also sensitive to the sample size of species occurrence records [41], and spatial sample selection bias [3].

The aim of this study was to evaluate the impact of sample size on model predictive performance for *D. dendriticum* in Italy, using a historical longitudinal data set of diagnostic data, and to evaluate the utility of opportunistic diagnostic data at a higher temporal resolution for predicting the distribution of this species.

## Materials and methods

### Overview

A Random Forest (RF) species distribution modelling algorithm was applied to *D. dendriticum* diagnosis data and environmental covariates to predict the spatial probability distribution of this species in Italy. The model was replicated using random subsets of the occurrence dataset to determine the sample size threshold below which model performance deteriorates. Models developed using annual subsets of occurrence data were compared against this threshold to evaluate the impact of using historic datasets with restricted sample size and temporal resolution on model performance.

Model development is documented according to the ODMAP guidelines for reporting Species Distribution Models ([42]; Appendix).

### Data

The preparation of both the covariate and the disease data was conducted in R, version 3.4.3 [39]. VECMAP^®^ was used to generate a basic presence-absence map for rapid visualisation of the georeferenced presence and absence of the parasites over the years.

#### *D. dendriticum* occurrence data

**Figure 2:**
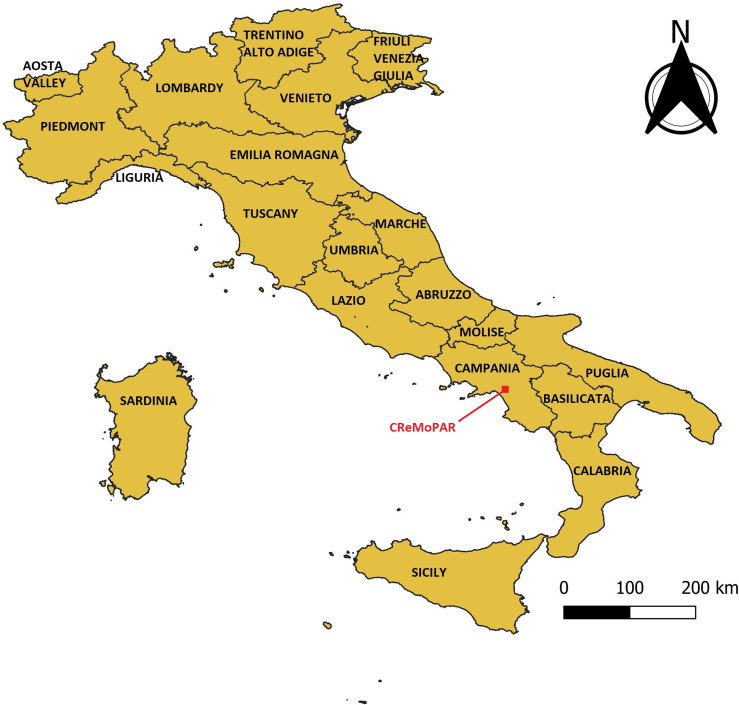
Administrative regions of Italy referred to in the paper.

The study area for this research is Italy, which is divided in 20 regions (Fig. 2) and has a surface area of 301.340 km^2^. Its climate varies depending on the region. Overall low precipitation is seen and the mean temperatures vary between 5 and 20 °C depending on the region (http://koeppen-geiger.vu-wien.ac.at/).

*D. dendriticum* data covering most of Italy ranging from 1999 to 2018 were provided by the Regional Centre for Monitoring of Parasitosis (CREMOPAR), Campania Region, Southern Italy. A wide variety of sources contributed to the historical data set, including scattered samples collected by veterinary practitioners, passive surveillance, and clustered data collected as part of active surveillance programmes in limited areas. None of these data sources were free from bias (e.g. samples from veterinarians were biased towards symptomatic cases) and most of the surveys were conducted in southern Italy due to the continuous monitoring service offered by the Department of Agriculture of the Campania Region, through the activities of CREMOPAR.

#### Environment covariates

In total, the predictor set used to develop the *D. dendriticum* distribution models comprised 39 variables (Table 1). Collinearity was checked using the variance inflation factor and Spearman rank correlation. Variables with VIF > 10 were removed from further analyses. Only one of variable pairs with correlation >0.7 was retained.

**Table 1:** Environmental co-variates (predictor data).

Abbreviation	Variable
NDVI_14A0	Normalised difference vegetation index transformed Fourier analysis band 14 – A0 – mean
NDVI_14A1	Normalised difference vegetation index transformed Fourier analysis band 14 – A1 – amplitude of annual cycle
NDVI_14A2	Normalised difference vegetation index transformed Fourier analysis band 14 – A2 – amplitude of bi-annual cycle
NDVI_14A3	Normalised difference vegetation index transformed Fourier analysis band 14 – A3 – amplitude of tri-annual cycle
NDVI_14D1	Normalised difference vegetation index transformed Fourier analysis band 14 – D1 – variance in annual cycle
NDVI_14D2	Normalised difference vegetation index transformed Fourier analysis band 14 – D2 – variance in bi-annual cycle
NDVI_14D3	Normalised difference vegetation index transformed Fourier analysis band 14 – D3 – variance in tri-annual cycle
NDVI_14DA	Normalised difference vegetation index transformed Fourier analysis band 14 – DA – combined variance in annual, bi-annual, and tri-annual cycles
NDVI_14MN	Normalised difference vegetation index transformed Fourier analysis band 14 – MN – minimum
NDVI_14MX	Normalised difference vegetation index transformed Fourier analysis band 14 – MX – maximum
NDVI_14P1	Normalised difference vegetation index transformed Fourier analysis band 14 – P1 – phase of annual cycle
NDVI_14P2	Normalised difference vegetation index transformed Fourier analysis band 14 – P2 – phase of bi-annual cycle
NDVI_14P3	Normalised difference vegetation index transformed Fourier analysis band 14 – P3 – phase of tri-annual cycle
NDVI_14VR	Normalised difference vegetation index transformed Fourier analysis band 14 – VR – variance in raw data parameter Fourier variable image values
BIO 1	Annual mean temperature (°C)
BIO 2	Annual mean diurnal range (°C)
BIO 3	Isothermality (°C)
BIO 4	Temperature seasonality (standard deviation) (°C)
BIO 5	Tmax of warmest month (°C)
BIO 6	Tmin of coldest month (°C)
BIO 7	Annual temperature range (°C)
BIO 8	Mean temperature of wettest quarter (°C)
BIO 9	Mean temperature of driest quarter (°C)
BIO 10	Mean temperature of warmest quarter (°C)
BIO 11	Mean temperature of coldest quarter (°C)
BIO 12	Annual precipitation (mm)
BIO 13	Precipitation of wettest month (mm)
BIO 14	Precipitation of driest month (mm)
BIO 15	Precipitation seasonality (coefficient of variation) (%)
BIO 16	Precipitation of wettest quarter (mm)
BIO 17	Precipitation of driest quarter (mm)
BIO 18	Precipitation of warmest quarter (mm)
BIO 19	Precipitation of coldest quarter (mm)
TempXX_A0	Temperature of XX (depending on the year that is modelled 09, 12, 13, 14, 15, 16) – amplitude of annual cycle
TempXX_A1	Temperature of XX (depending on the year that is modelled 09, 12, 13, 14, 15, 16) – amplitude of bi-annual cycle
TempXX_A2	Temperature of XX (depending on the year that is modelled 09, 12, 13, 14, 15, 16) – amplitude of tri-annual cycle
TempXX_P0	Temperature of XX (depending on the year that is modelled 09, 12, 13, 14, 15, 16) – phase of annual cycle
TempXX_P1	Temperature of XX (depending on the year that is modelled 09, 12, 13, 14, 15, 16) – phase of bi-annual cycle
TempXX_P2	Temperature of XX (depending on the year that is modelled 09, 12, 13, 14, 15, 16) – phase of tri-annual cycle

Monthly normalised difference vegetation index (NDVI) data with a resolution of 1 km, a vegetation index which measures the photosynthetic activity of plants, were obtained from MODerate-resolution Imaging Spectroradiometer (MODIS) imagery (http://modis.gsfc.nasa.gov/). These data were Fourier transformed using the methods of [13], to derive biologically-relevant secondary variables. We used this variable to help identify the habitat of *D. dendriticum*, as mentioned above.

BioClim variables [17] at a resolution of 30 s, were added to the set of environmental predictors. These are climate indicators that may affect species distribution, summarising the period 1970–2000, and are developed by the U.S. Geological Survey (USGS). They represent information regarding annual and seasonal conditions and differences through the different seasons in one year. This can be as a derived variable or in a time-series [30]. These data were used for the baseline model and sample size evaluation.

To fit models to annual data, ERA5 temperature data for the corresponding year were used to derive bioclimatic summaries to replace these variables of the BioClim (Bio01–Bio11) data set in the individual year models. ERA5 was developed by the European Centre of Medium-Range Weather Forecast (ECMWF) in 2017, including hourly estimates of different variables. It contains information such as temperature, humidity, pressure and wind in the specific year of interest [1].

Host distribution data were obtained from the Gridded Livestock of the World (GLW 3) database, a collaboration of the Food and Agriculture Organisation (FAO) and Environmental Research Group Oxford (ERGO). This database provides the distribution layers of bovines, small ruminants, pigs, and poultry derived by multivariate regression [33].

#### Spatial bias-correction

The occurrence data set did not include data points throughout Italy and is therefore not representative for the whole of Italy. As a result, we masked out the parts of Italy for which the data are not representative using an environmental envelope, also called climatic envelope, which is based on a set of environments in which it is supposed that the species persist, because the environmental needs of the species are satisfied [40]. For this, Mahalanobis distance (MD) was used. Farber and Kadmon [16] showed that using MD resulted in more accurate predictions of species distributions compared to standard envelopes that are rectilinear. Hereafter, only the area within this environmental envelope was used for model development and mapping, to avoid projecting outside of the range of the model input data.

#### Data extraction

Prior to model development, data were first prepared in VECMAP^®^ by extracting environmental covariate data from the sites where *D. dendriticum* is present and absent using the “Extract data” function. The extract data tool iterates through all the defined environment covariate images in the predictor suite and extracts an environmental value for each data occurrence data point used to develop (train) the model, and data outliers are excluded based on the standard deviation of the predictor values. Second, the extracted data set needs to be balanced when using non-bootstrapping models such as Random Forest. We randomly sampled the largest class to result in an equalisation of the presence and absence points.

### Model development

#### Random Forest baseline model – best case scenario (BCS)

A machine learning modelling technique, Random Forest (RF), was applied using VECMAP^®^ software, which is based on the randomForest R package [24]. This modelling technique was previously used for modelling bulk-milk tank antibodies against liver fluke at a European scale [10]. First, we computed a baseline Random Forest model using the entire occurrence and environmental covariate data set. The parasitic infection (presence/absence of the parasite in the final host) was used as a proxy for the parasite. The random Forest algorithm was applied to the extracted data (Sect. Data extraction) to group the presence-absence data into clusters based on different eco-climatic patterns using a recursive partitioning approach (similar to a decision tree). This allows recognition of any pattern in the data [10].

Initially, a model was fitted to the complete dataset (all occurrence and covariate data), specifying 500 replicate trees and 8 environmental variables to be selected at random at each node. Variable importance was then assessed using mean decrease accuracy and mean decrease Gini and a reduced set of 3 environmental variables selected based on their importance (cf. Tables 2 and 3). A second model was then fitted to the reduced set of environmental variables, specifying 100 replicate trees and 6 variables to be selected at random at each node. Model evaluation is based on standard model statistics. These include sensitivity, specificity, Cohen’s kappa, and area under curve (AUC). Expert analysis is also used to evaluate the plausibility of the mapped model outputs.

**Table 2:** Statistical model output baseline model using RF of *Dicrocoelium dendriticum*.

	BCS	−10%	−20%	−30%	−40%	−50%	−60%	−70%	−80%	−90%
Presence points	2508	2258	2008	1758	1508	1258	1008	758	508	258
Kappa	0.61	0.56	0.54	0.60	0.53	0.56	0.54	0.48	0.53	0.54
AUC	0.72	0.71	0.71	0.71	0.72	0.70	0.70	0.67	0.71	0.71
Sensitivity	0.68	0.68	0.66	0.69	0.67	0.64	0.64	0.62	0.66	0.70
Specificity	0.64	0.64	0.63	0.63	0.65	0.63	0.65	0.60	0.65	0.61
Predictor importance	Bio11	Bio11	Bio11	Bio05	Bio01	Bio09	Bio01	Bio11	Bio11	Bio01
	Bio05	Bio08	Bio01	Bio11	Bio09	Bio11	Bio09	Bio05	Bio01	Bio10
	Bio07	Bio06	Bio12	Bio06	Bio11	Bio16	Bio11	Bio01	Bio06	Bio05

**Table 3:** Statistical model output using RF of *Dicrocoelium dendriticum*: BCS and −70% of baseline model compared to statistical output of 2009, 2012, 2013, 2015 and 2016.

	BCS	−70%	2009	2012	2013	2015	2016
Presence points	2508	758	175	163	134	120	415
Kappa	0.61	0.48	0.47	0.59	0.48	0.56	0.47
AUC	0.72	0.67	0.65	0.74	0.67	0.73	0.62
Sensitivity	0.68	0.61	0.60	0.67	0.57	0.70	0.61
Specificity	0.64	0.60	0.62	0.69	0.62	0.64	0.60
Predictor importance	Bio11	Bio11	Bio16	Bio12	Bio12	Bio12	Bio13
	Bio05	Bio05	Bio13	Bio16	Bio18	Bio16	Bio19
	Bio07	Bio01	Bio15	Bio19	Bio16	Bio14	Bio12

#### Minimal occurrence data sample size

The minimal number of occurrence data points needed to build a stable model was determined by first starting with the best-case scenario (BCS; described above). This is the maximum number of data points that can be used based on the number of presence and absence points available to assemble a baseline model. Thereafter, RF model replicates were developed as described above, with the occurrence data incrementally reduced by subtracting 10% of the total number of samples at random with one replicate. Model performance statistics were then compared between replicates; when a plateau face was reached in the statistical output, this was the minimal sample size needed to build a stable model.

#### Model performance based on annual historic data

We then investigated the possibility of developing annual distribution maps using only the data available for each single year for which data were available. Again, these models were computed using the random Forest machine learning algorithm of the VECMAP^®^ software package, and the statistical performance compared against the BCS model and the model using the minimal sample size.

## Results

### Data exploration

**Figure 3:**
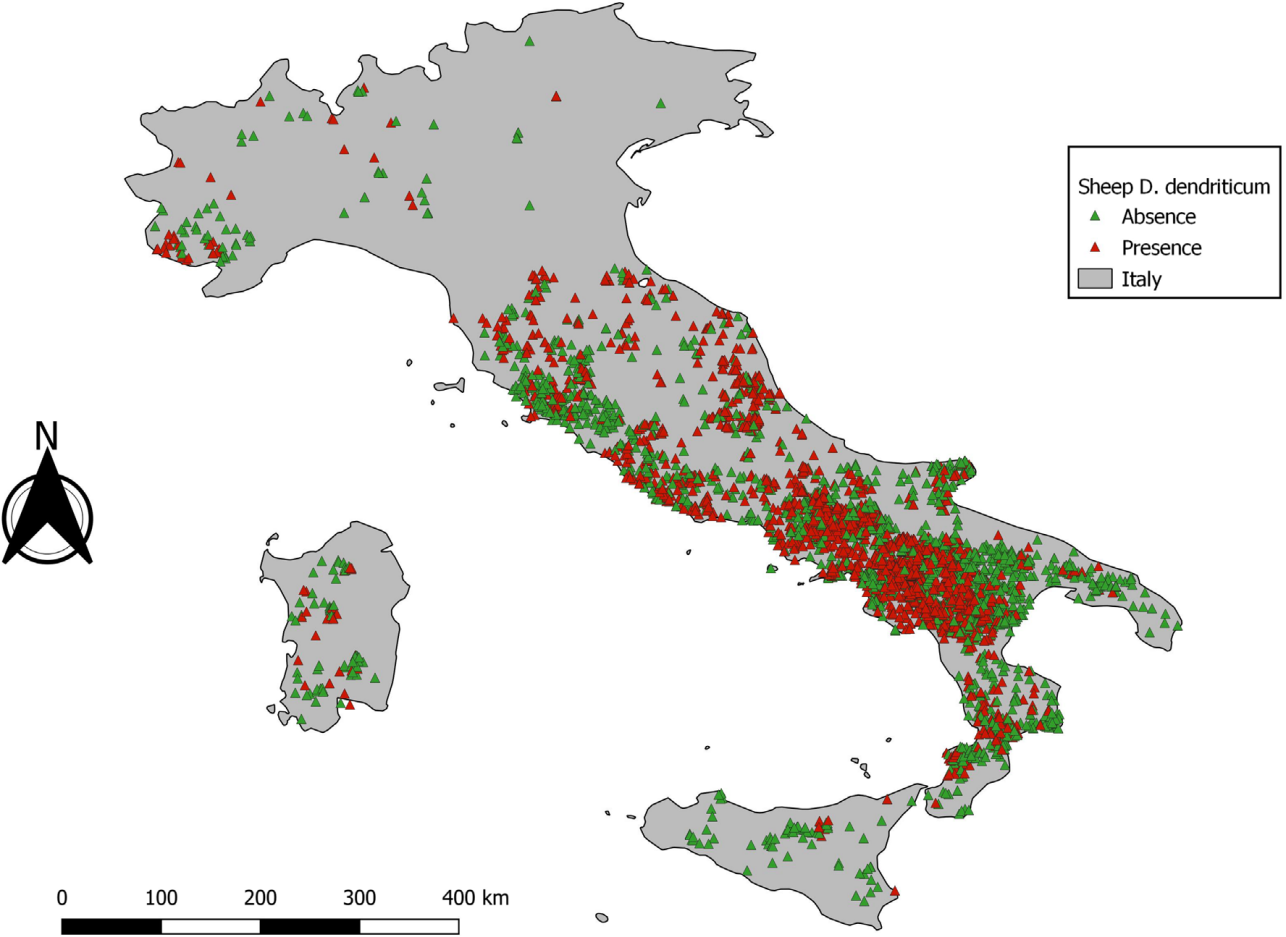
Data distribution for *Dicrocoelium dendriticum* from 1999–2018.

A total of 5131 *D. dendriticum* occurrence records were available. The distribution of *D. dendriticum* occurrence records was not spatially homogeneous (Fig. 3). Most of the data were clustered in central and southern Italy, specifically in the Campania region and its neighbouring regions (bias due to the location of CREMOPAR). The five years with the most presence points were: 2016 (415), 2009 (305), 2006 (184), 2008 (169), and 2012 (165). After removal of outliers, the five years with the highest sample size for the best-case scenario, the maximal number of samples that can be used to model were: 2016 (415), 2009 (178), 2013 (135), 2012 (165), and 2015 (120).

### Baseline model – best case scenario (BCS)

**Figure 4:**
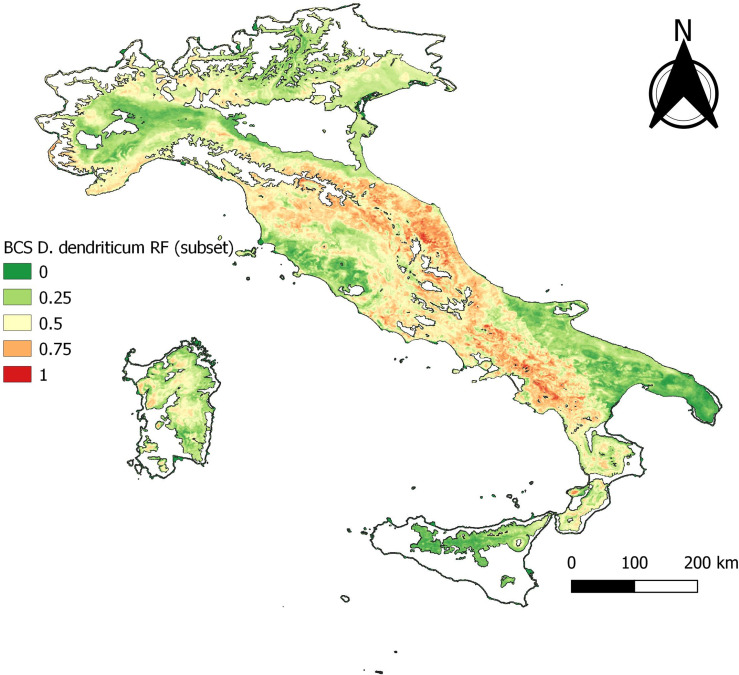
Baseline model, BCS.

Following initial RF model fitting, a reduced variable set (marked with an asterisk in Table 1) was used for development of subsequent models. Using this reduced variable set, the BCS model predicted an elevated probability of occurrence of *D. dendriticum* throughout Campania, Calabria, Lazio, Abruzzo, Marche, and Emilia-Romagna regions (Figs. 2 and 4, Table 2). This reflects the known distribution of *D. dendriticum* on a broad spatial scale.

### Minimal occurrence data sample size

**Figure 5:**
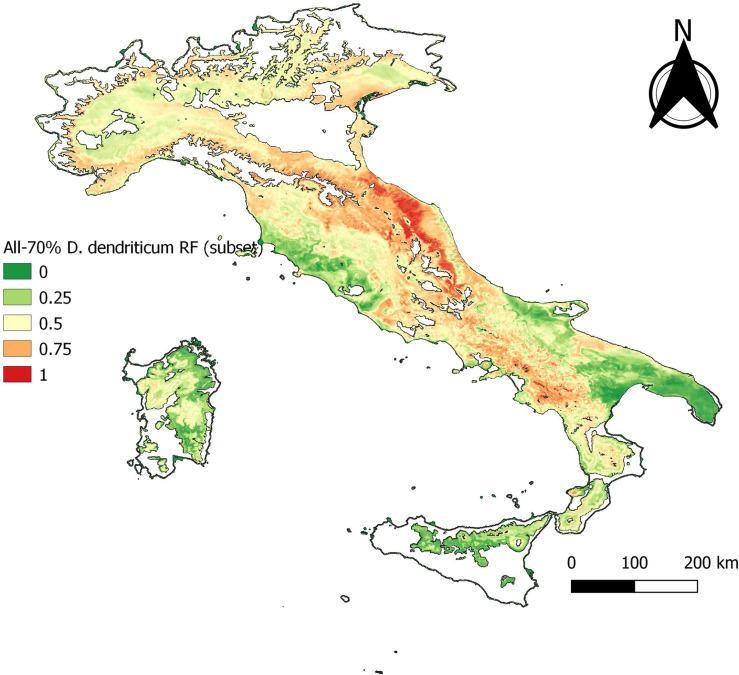
Baseline model, −70%.

The RF model outputs are given in Table 2. A clear drop of all statistics is observed at −70% of the data points. This is referred to hereafter as the “cut-off” and corresponds to 758 presence- and 758 absence samples. The “cut-off” model using this reduced dataset of 30% of the occurrence data (Fig. 5) yielded similar spatial predictions to the BCS model, with the exception of a slightly elevated risk in Marche and Emilia-Romagna (Fig. 4, cf. Fig. 5).

With the exception of Bio11 (Mean Temperature of Coldest Quarter), which was identified as one of the 3 most important variables in all models except the model using only 10% of the occurrence data, variable importance varied according to the occurrence data subset used (Table 2).

### Model performance based on annual historic data

No individual year reached the cut-off value of 758 presence samples. Therefore, we decided to model the 5 years that contained the highest number of data: 2009 (*n* = 178), 2012 (*n* = 165), 2013 (*n* = 135), 2015 (*n* = 120), and 2016 (*n* = 415). Model performance and variable importance varied according to the occurrence data subset used (Table 3). Fully mapped details were provided only for 2012 and 2016, the most interesting input for a discussion, in order to avoid overloading this paper.

**Figure 6:**
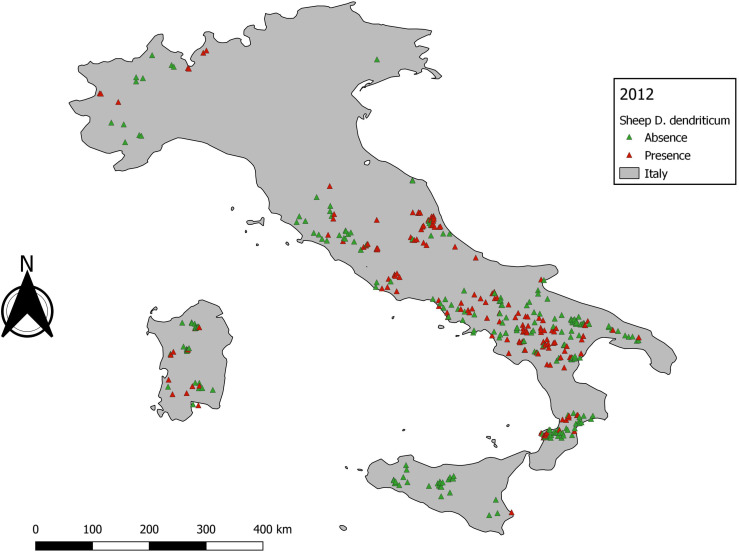
Data distribution for *Dicrocoelium dendriticum* 2012.

**Figure 7:**
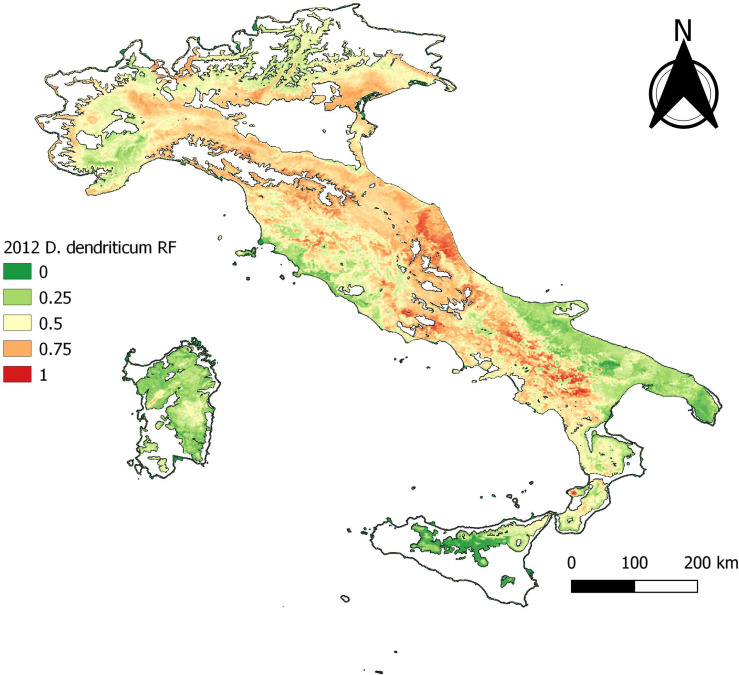
Annual distribution model 2012.

**Figure 8:**
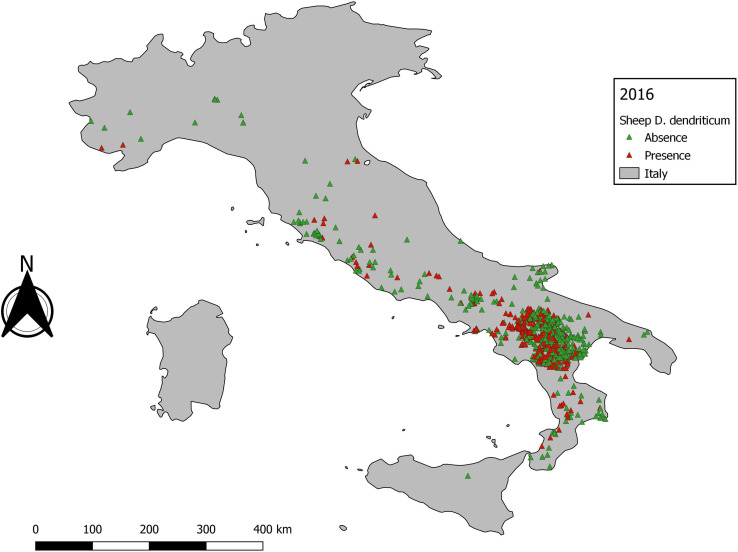
Data distribution for *Dicrocoelium dendriticum* 2016.

**Figure 9:**
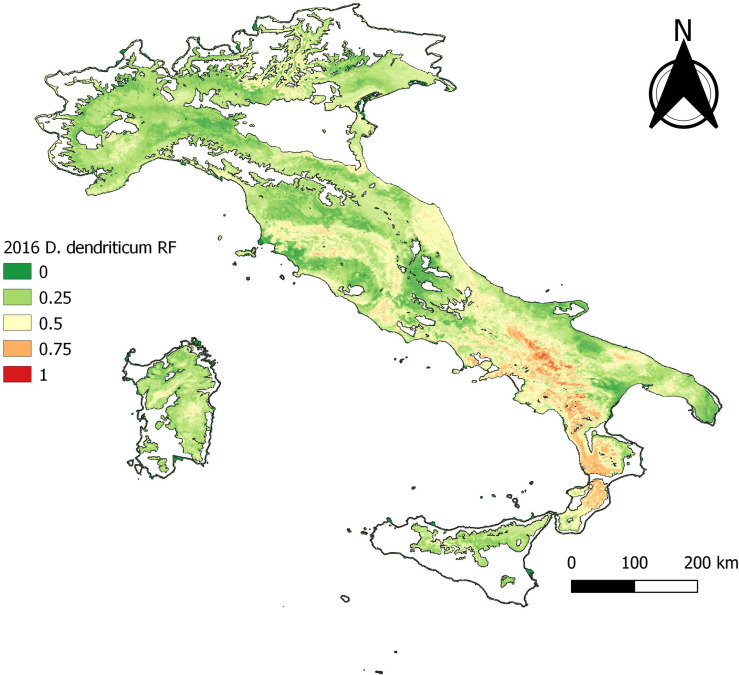
Annual distribution model 2016.

The statistical output of 2012 with 165 presence data samples, showed that overall higher values than the cut-off are observed. The statistical output is almost equal to the baseline model (Table 3). The data exploration map of 2012 (Fig. 6) showed that most of the data are located in the Campania and Basilicata regions. The model output of 2012 (Fig. 7) broadly reflects the BCS model (Fig. 4), predicting 0.5–1 probability of presence zones from the Po valley down to Calabria. Puglia, Sicily, and Sardinia are low-prediction regions. The statistical output of 2016 (Table 3), 415 samples, showed lower values compared to the baseline model. Except for AUC, the statistical values are approximately equal to the cut-off. The data exploration map of 2016 (Fig. 8) showed that most of the data are located in the Campania and Basilicata regions. A clustered zone of samples can also be observed in these regions. Despite the good statistical performance, the central-northern regions which have previously reported *D. dendriticum* infections (Fig. 3), and were previously identified as having elevated probability of presence using the BCS model (Fig. 4), are predicted to have a low probability of presence using only 2012 data. Compared to the other individual year models an overall low prediction zone is predicted throughout whole Italy in 2016 (Fig. 9). In Calabria and parts of Campania and Basilicata 0.75 predicted probability of presence zones are observed.

The results obtained for the other three years (maps not shown here) are summarised below.

The statistical output of 2009 (Table 3), with 178 samples, showed that compared to the statistical output of the cut-off (−70%), the values are approximately equal but still lower than the baseline model. The data exploration map of 2009 shows that most of the presence/absence points are located in the Campania and Basilicata regions. In the model output of 2009, we can observe 0.25–0.5 presence zones throughout Italy. In Marche, 0.75 predictive zones are observed and in Campania and Basilicata, high prediction (0.75–1) zones are observed.

The statistical output of 2013 (Table 3), 135 samples, showed that overall, except for Kappa, the statistical values are in the same range as the baseline model. The data exploration map of 2013 shows that most of the data are located in the Campania and Basilicata regions. Compared to 2012, the same pattern is seen in presence prediction but an overall higher prediction rate is observed. Marche is predicted as a high (1) presence region. Parts of Apulia, Sicily, and Sardinia had lower (0.25–0.5) prediction zones.

The statistical output of 2015 (Table 3), 120 samples, showed that the statistical values are almost equal to the baseline model. The data exploration map of 2015 shows that most of the data are located in the Campania and Basilicata regions. Again, the same pattern is observed in the model output starting from the Po valley to Calabria, not including the main part of Puglia and Sicily. Sardinia has a higher prediction zone (0.5–0.75) compared to the model from 2013. A higher prediction zone is seen around Marche, compared to 2013.

## Discussion

The first step in the spatial modelling process is planning and gathering a presence-absence data set. In this research, a historical data set for Italy covering the period from 1999 to 2018 was provided by CREMOPAR. The data were obtained using both active and passive surveillance. The advantages of having access to such a large data set are obvious, but one of the main disadvantages is that the data were not specifically collected for developing spatial models. It seems clear that this results in a sampling bias: (i) some areas are oversampled (especially in central and southern regions); and (ii) there are large geographical gaps. If this bias is not corrected the spatial distribution model (SDM) outputs may display the sampling effort rather than the real species distribution [38]. In this study, this issue was partially solved by computing a mask based on the environmental envelope of *D. dendriticum* that excluded the pixels that were not representative for the sampled pixels which will be discussed later in this section.

The second step is data exploration. An important observation when exploring the occurrence data of dicrocoeliosis was a clustering of samples in some areas of southern Italy. The reason for this is probably mainly the active monitoring programme carried out by CREMOPAR. As a result, the area surrounding the CREMOPAR is overrepresented as compared to other sampled areas. This should not be confused with a clustered distribution pattern of this parasite, as reported in other studies [4, 11, 29] where clustered areas of presence were observed within larger sampled, but negative, areas. In these surveys, the clustering observed was likely due to the specific eco-climatic condition required by intermediate hosts of *D. dendriticum* to develop.

When making exploratory models, it is important to first determine the minimal sample size in order to model a reliable predictive map. Mateo et al. [26] showed that generated models are influenced by sample size and prevalence. The predictive power of a model increases when information is added. This applies until the statistical values reach a “plateau”. From then, model performance is not considerably enhanced when additional data are added. When sample size decreases, the accuracy, reliability, and stability of the model should decrease as well. Therefore, it can be concluded that in order to generate a robust model a minimum sample size, and more specifically a minimum number of presence points, is needed. Also, SDMs are used to limit the sampling effort. Hence, setting a minimum sample size allows production of precise SDMs, without wasting expensive resources [37]. No individual annual subset of the occurrence data reached the cut-off determined from the baseline models’ statistics. Therefore, the top 5 of most samples throughout the individual years were selected to do further modelling as a proof of concept.

### Baseline model

For this research, we aimed to model the presence/absence of the parasite detected in the final host, sheep, using eco-climatic environmental predictor data. These are expected to show the strongest relationship with the distribution of the intermediate host and free-living stages of the parasite. We hence used predictor data that indirectly influence infection presence. In this case, an absence of data means that the infection did not occur when the diagnosis was made. Therefore, more presence data are needed to model a reliable predictive map. Hendrickx [20] showed that separately modelling a vector, a disease and its main symptom (e.g. anaemia) using eco-climatic predictor data, systematically resulted in a lower level of accuracy of the disease model for the same number of samples. This was explained by the fact that other factors than climatic data affect the distribution of a disease in a host, and that many different diseases may affect an observed symptom such as anaemia.

Indirect measures of parasite presence were used in this study as a proxy for the presence of the parasite. Copromicroscopic analysis for the presence of parasite eggs is the most widely used diagnostic procedure for *D. dendriticum* and other helminths [9], where accurate detection of the parasite in the environment is difficult (e.g. liver fluke metacercariae on pasture), and more direct measures of parasite presence in the host are invasive (e.g. post mortem to confirm parasite presence). Copromicroscopic techniques are advantageous as they allow for larger sample sizes than would be possible with more invasive or laborious sampling methods. However, these approaches may produce false-negative results where patent infections are not always detectable. In our case, we used eco-climatic data to model infection data, whilst other factors such as farm management and the presence of the intermediate host will also affect it. It is also very likely that disease management strategies may vary greatly over such a wide geographical range. The fact that these could not be included as co-variates may affect the quality of the model outputs. Nevertheless, the developed models performed well both statistically and qualitatively, showing that whilst not including such co-variate data may affect the reliability of identifying causal factors, this does not necessarily affect the efficiency of pattern matching that this type of modelling implements. The models therefore provide a good basis for further exploration of sample size requirements and the impact of sample subset on model performance.

The produced model output using the entire observed presence data set yielded an overall satisfactory result. In both the southern and central parts of Italy, the model provides satisfactory spatial detail as a valid tool for further field work towards refining the knowledge about the distribution patterns of this parasite. The lack of information in the most northern part of Italy is partially solved by removing non-representative areas from the modelling process.

Interestingly, when reducing the sample size (−70% model), whilst the general distribution pattern remains the same, this results in a loss of spatial detail. Here, the southern third of Italy remains very similar to the full model output, in the central part the high-risk (high probability of presence) areas increase in size, and in the northern third, there is also a strong shift towards a higher category. This suggests that the negative effect of an uneven spatial distribution mainly affects smaller sample sizes, as less variation within a sample may reflect less variation in the output.

### Annual distribution models

When developing models for individual years, the Bioclim data for temperature (from Bio01 to Bio11) were replaced with data derived from ERA5 for this variable. ERA5, developed in 2017, provides more precise data because the data are registered hourly [1] compared to Bioclim, developed in 2005, that provides monthly climatic data based on long-term averages (1970–2000) [21]. This allows us to take into consideration specific climatic conditions prevailing in each year.

The individual year models confirm the statement that sample size affects model performance [26]. Though no individual year reached the sample size cut-off and results for annual models were variable, the quality of the outputs obtained for the years with the highest number of observations is encouraging. The output statistics differ for each year, but there is no clear decrease or increase in the statistics. The best prediction rate and statistical output were obtained for 2012 (Fig. 7) and the worst for 2016 (Fig. 9), even though the former sample size was significantly smaller than the latter. This can be explained by looking at the distribution of *D. dendriticum* in each individual year. The statistics of 2012 (Table 3) are improved compared to the cut-off (−70%) model. The mapped model output (Fig. 7) shows a similar pattern for southern and central Italy. For this model, the input data (Fig. 6) are more evenly geographically distributed throughout Italy, and there is no dense data cluster in the southern third of the country. The statistics for 2016 (Table 3) are worse compared to the cut-off. For this year, a major portion of the data were strongly clustered in the southern part of Italy (Fig. 8). The mapped model output also shows a very different spatial distribution pattern in the central and northern parts of Italy; this is unlikely to occur because of climatic differences between years. As a result, the 2016 model failed to identify the central and northern region of Italy as suitable for *D. dendriticum.* This may also be affected by our choice to use the same full-data suitability mask when computing the annual models. In future work, we will further test the effect of using different suitability masks for each year.

Syfert et al. [38] showed that the prediction accuracy of models made with spatially clustered data is inferior to that with models which are not clustered. To overcome this, it may be possible to filter the database to reduce spatial autocorrelation, resulting in a data set with, for instance, maximum one record per km^2^ cell. The impact of selecting maximum or mean values per pixel will be explored in further work. However, in this case, this would not solve the issues that (a) no year made the cut-off of minimal sample size to model a sufficiently stable predictive model, and (b) geographical gaps in the data set are too large.

Appropriate knowledge and robust experience on a parasite and its intermediate hosts are required to interpret data sets for their suitability when modelling. The open availability of many species occurrence datasets (e.g. https://www.gbif.org) and the apparent ease of implementing basic species distribution models, make species distribution modelling attractive, without considering the quality of the data used to develop such models. Our results simultaneously highlight the potential opportunities for modelling parasite distributions using longitudinal datasets of indirect measures of presence (diagnostic data), and the limitations of highly clustered data with a limited temporal range. Given the weaknesses of our data set, discussed above, the obtained results suggest that the proposed approach may contribute to highlight differences between years provided that the input data set is more evenly geographically distributed, and that additional predictor variables reflecting non-environmental factors, such as farm management, affecting the presence of the infection, are identified and available at sufficient spatial detail.

In conclusion, the spatial distribution of the input data appears to be more important than the actual sample size when computing species distribution models. This is often a major issue when using historical data for developing spatial models. Such data sets often include sampling biases and large geographical gaps. If this bias is not corrected, the SDM outputs may display the sampling effort rather than the real species distribution.

## Appendix

**ODMAP checklist:** Hendrickx et al., 2020: Constraints of using historical data for modelling the spatial distribution of helminth parasites in ruminants.

ODMAP element	Contents
Overview	
Authorship	**Authors:** Hendrickx A, Marsboom C, Rinaldi L, Rose Vineer H, Morgoglione EM, Sotariki S, Cringoli G, Claerebout E and Hendrickx G.
	**Contact email:** ghendrickx@avia-gis.com
	**Title:** Constraints of using historical data for modelling the spatial distribution of helminth parasites in ruminants.
	**DOI:**
Model objective	**Objective:** Explanation < Mapping (Inference < Interpolation).
	**Target outputs:** Maps of relative probability of presence for Italy.
Taxon	Parasitic helminth, *Dicrocoelium dendriticum*.
Location	Italy.
Scale of analysis	**Spatial extent (Lon/Lat):** 6.7–18.5 °E, 36.6–47.12 °N.
	**Spatial resolution:** 1 km.
	**Temporal extent/time period:** 1999–2018.
	**Type of extent/boundary:** Administrative boundary (Italian border).
Biodiversity data overview	**Observation type:** Veterinary diagnostic data and field survey.
	**Response/data type:** Presence–absence.
Type of predictors	Vegetation, bioclimatic, livestock density.
Conceptual model/hypothesis	**Hypotheses about species-environment relationships:** There is evidence that the distribution of *D. dendriticum* is driven by vegetation and climate (both directly and indirectly via intermediate host influences). However, the quality, temporal resolution and quantity of available occurrence data may constrain the application of species distribution models to predict the distribution of *D. dendriticum* cases. We developed SDMs to evaluate the impact of subsetting historic occurrence data on model performance.
Assumptions	**We assumed that:** Diagnostic data were representative of presence or absence of infection in the host. Sensitivity of diagnostic data does not change in space or time. The chosen environmental covariates represent all relevant environmental drivers of distribution. The data encompass the species’ realised niche in the area modelled (after bias-correction – see below). Sample selection bias is adequately corrected (see below).
SDM algorithms	**Algorithms:** Random Forest – this method was chosen because of experience with the model and good performance in previous modelling exercises.
	**Model complexity**: 500 trees with 8 variables.
	**Ensembles:** Not applicable (except for bagging implicit in the Random Forest algorithm).
Model workflow	After preparation of environmental covariates, removal of errors and bias-correction (see below), Random Forest models were fitted to the full dataset, and to a reduced set of covariates identified as important in the full model. This process was repeated for incrementally increasing sample sizes (see portioning information below) to identify the minimal sample size, below which statistical performance deteriorates. Models were also fitted using the same process to annual occurrence data.
Software	**Software:** Environmental variable processing was completed in R v3.4.3 (R Core Team, 2017 [43]). Mapping and Random Forest modelling were completed in VECMAP^®^ (https://www.avia-gis.com/vecmap).
Data	
Biodiversity data	**Taxon names:***Dicrocoelium dendriticum*.
	**Ecological level:** Species.
	**Data source:** CReMoPAR, a parasitological reference lab from the Naples (Italy) area. Diagnostic data collected 1999–2018.
	**Sampling design:** Samples submitted from throughout Italy for diagnosis (faecal egg counts), opportunistic samples collected in the region surrounding CReMoPAR.
	**Sample size:** 5131 occurrences.
	**Regional mask**: Data were clipped to the Italian boundary.
	**Scaling:** Not applicable.
	**Background data:** Not applicable.
	**Errors and biases**: Parts of Italy not represented in the dataset were masked using an environmental envelope generated using a Mahalanobis distance approach. The area within this environmental envelope was used for model development to avoid projecting model predictions outside of the range of the occurrence data. The data set was balanced by randomly subsampling the largest class.
Data partitioning	A model was developed using the full datasets to demonstrate the “best-case scenario” (BCS), before reducing the size of occurrence dataset in 10% increments at random, to evaluate the impact of sample size on model performance.
	Models were also fitted to data for the 5 years between 1999 and 2018 with the highest occurrence data sample size to evaluate the impact of dataset on model performance.
Predictor variables	**Predictor variables and data sources:** NDVI data from MODIS (http://modis.gsfc.nasa.gov/), Fourier-transformed according to the methods described by Estrada-peña et al. [13]. Bioclimatic variables [17] derived from ERA5 [1] temperature data. Gridded Livestock of the World livestock density data [33].
	**Spatial resolution and extent of the raw data:** The livestock density data were available at a 10 km resolution. Bioclimatic data were available at a 1 km resolution. NDVI data were available at a 1 km resolution.
	**Geographic projection:** WGS84.
	**Temporal resolution and extent of the raw data:** The livestock density data represent predicted livestock density for 2011. Bioclimatic variables and NDVI data were averaged for the temporal extent of the occurrence dataset (1999–2018).
	**Data processing:** The extent of all data were clipped to the Italian boundary before processing. Resampling/aggregation was not performed to standardise resolution.
Model	
Variable pre-selection	The choice of initial covariates was made as a compromise between availability and ecological/biological relevance to the study species. Only weakly correlated covariates were included in the models.
Multicollinearity	Multicollinearity between the covariates was investigated using the variance inflation factor and Spearman rank correlations. Covariates with VIF > 10 were discarded. Only one variable from pairs with correlations >0.7 was retained to avoid model overfitting.
Model settings	Default settings were used throughout, except for the number of replicates and the number of variables to evaluate at each node. For initial models using all variables (variable selection step), 500 replicates and 8 variables were specified. For models using the reduced set of variables selected for their importance, 100 replicates and 6 nodes were specified.
Model estimates	Covariate importance was estimated with mean decrease accuracy and mean decrease Gini.
Model averaging/ensembles	Not applicable.
Non-independence	Not done, see discussion.
Assessment	
Performance statistics	Model evaluation is based on standard model statistics. These include Sensitivity, Specificity, Cohen’s Kappa, and Area Under Curve (AUC).
Plausibility checks	Expert analysis is used to evaluate the plausibility of the mapped model outputs.
Prediction	
Prediction output	Predictions of relative probability of presence of *D. dendriticum* is expressed on a continuous scale. Maps are restricted to the environmental envelope identified using a Mahalanobis distance approach (see above) to avoid projecting outside of the range of the occurrence data.
Uncertainty quantification	Not applicable – ensembles not performed.

## References

[1] Albergel C, Dutra E, Munier S, Calvet JC, Munoz-Sabater J, De Rosnay P, Balsamo G. 2018. ERA-5 and ERA-Interim driven ISBA land surface model simulations: Which one performs better? Hydrology and Earth System Sciences, 22(6), 3515–3532.

[2] Arbabi M, Nezami E, Hooshyar H, Delavari M. 2018. Epidemiology and economic loss of fasciolosis and dicrocoeliosis in Arak, Iran. Veterinary World, 11(12), 1648. 30774253 10.14202/vetworld.2018.1648-1655 PMC6362328

[3] Beck J, Böller M, Erhardt A, Schwanghart W. 2014. Spatial bias in the GBIF database and its effect on modeling species’ geographic distributions. Ecological Informatics, 19, 10–15.

[4] Bennema S, Vercruysse J, Claerebout E, Schnieder T, Strube C, Ducheyne E, Hendrickx G, Charlier J. 2009. The use of bulk-tank milk ELISAs to assess the spatial distribution of *Fasciola hepatica*, *Ostertagia ostertagi* and *Dictyocaulus viviparus* in dairy cattle in Flanders (Belgium). Veterinary Parasitology, 165(1–2), 51–57. 19656630 10.1016/j.vetpar.2009.07.006

[5] Bennema SC, Ducheyne E, Vercruysse J, Claerebout E, Hendrickx G, Charlier J. 2011. Relative importance of management, meteorological and environmental factors in the spatial distribution of *Fasciola hepatica* in dairy cattle in a temperate climate zone. International Journal for Parasitology, 41(2), 225–233. 20887726 10.1016/j.ijpara.2010.09.003

[6] Bosco A, Rinaldi L, Musella V, Pintus D, Santaniello M, Morgoglione M, Zacometti G, Cringoli G. 2013. Helminths in Sheep on Farms of the Basilicata Region of Southern Italy, in Trends in Veterinary Sciences, Boiti C, Ferlazzo A, Gaiti A, Pugliese A, Editors. Springer: Berlin, Heidelberg.

[7] Bosco A, Rinaldi L, Musella V, Amadesi A, Cringoli G. 2015. Outbreak of acute fascioliosis in sheep farms in a Mediterranean area arising as a possible consequence of climate change. Geospatial Health, 9(2), 319–324. 25826313 10.4081/gh.2015.354

[8] Colwell DD, Goater CP. 2010. Dicrocoelium dendriticum in cattle from Cypress Hills, Canada: Humoral response and preliminary evaluation of an ELISA. Veterinary Parasitology, 174(1–2), 162–165. 20817361 10.1016/j.vetpar.2010.08.004

[9] Cringoli G, Rinaldi L, Veneziano V, Capelli G, Scala A. 2004. The influence of flotation solution, sample dilution and the choice of McMaster slide area (volume) on the reliability of the McMaster technique in estimating the faecal egg counts of gastrointestinal strongyles and Dicrocoelium dendriticum in sheep. Veterinary Parasitology, 123(1), 121–131. 15265576 10.1016/j.vetpar.2004.05.021

[10] Ducheyne E, Charlier J, Vercruysse J, Rinaldi L, Biggeri A, Demeler J, Brandt C, de Waal T, Selemetas N, Höglund J, Kaba J, Kowalczyk SJ, Hendrickx G. 2015. Modelling the spatial distribution of *Fasciola hepatica* in dairy cattle in Europe. Geospatial Health, 9(2), 261–270. 25826307 10.4081/gh.2015.348

[11] Ekstam B, Johansson B, Dinnétz P, Ellström P. 2011. Predicting risk habitats for the transmission of the small liver fluke, *Dicrocoelium dendriticum* to grazing ruminants. Geospatial Health, 6(1), 125–131. 22109870 10.4081/gh.2011.164

[12] Elith J, Leathwick JR. 2009. Species distribution models: ecological explanation and prediction across space and time. Annual Review of Ecology, Evolution, and Systematics, 40(1), 677–697.

[13] Estrada-Peña A, Estrada-Sánchez A, de la Fuente J. 2014. A global set of Fourier-transformed remotely sensed covariates for the description of abiotic niche in epidemiological studies of tick vector species. *Parasites Vectors*, 7, 302. 24984933 10.1186/1756-3305-7-302 PMC4089935

[14] Ezatpour B, Hasanvand A, Azami M, Anbari K, Ahmadpour F. 2015. Prevalence of liver fluke infections in slaughtered animals in Lorestan. Iranian Journal of Parasitic Diseases, 39(4), 725–729. 10.1007/s12639-014-0428-4 PMC4675594 26688642

[15] Fairweather I, Brennan GP, Hanna REB, Robinson MW, Skuce PJ. 2020. Drug resistance in liver flukes. International Journal for Parasitology: Drugs and Drug Resistance, 12, 39–59. 32179499 10.1016/j.ijpddr.2019.11.003 PMC7078123

[16] Farber O, Kadmon R. 2003. Assessment of alternative approaches for bioclimatic modeling with special emphasis on the Mahalanobis distance. Ecological Modelling, 160(1–2), 115–130.

[17] Fick SE, Hijmans RJ. 2017. WorldClim 2: new 1-km spatial resolution climate surfaces for global land areas. International Journal of Climatology, 37(12), 4302–4315.

[18] González-Warleta M, Lladosa S, Castro-Hermida JA, Martínez-Ibeas AM, Conesa D, Muñoz F, López-Quílez A, Manga-González Y, Mezo M. 2013. Bovine paramphistomosis in Galicia (Spain): prevalence, intensity, aetiology and geospatial distribution of the infection. Veterinary Parasitology, 191(3–4), 252–263. 23022489 10.1016/j.vetpar.2012.09.006

[19] Gordon DK, Zadoks RN, Stevenson H, Sargison ND, Skuce PJ. 2012. On farm evaluation of the coproantigen ELISA and coproantigen reduction test in Scottish sheep naturally infected with *Fasciola hepatica*. Veterinary Parasitology, 187(3–4), 436–444. 22421492 10.1016/j.vetpar.2012.02.009

[20] Hendrickx G. 1999. Georeferenced decision support methodology towards trypanosomosis management in West Africa. Ghent, Belgium: Universiteit Gent.

[21] Hijmans RJ, Cameron SE, Parra JL, Jones PG, Jarvis A. 2005. Very high resolution interpolated climate surfaces for global land areas. International Journal of Climatology, 25(15), 1965–1978.

[22] Jeandron A, Rinaldi L, Abdyldaieva G, Usubalieva J, Steinmann P, Cringoli G, Utzinger J. 2011. Human infections with *Dicrocoelium dendriticum* in Kyrgyzstan: the tip of the iceberg? Journal of Parasitology, 97(6), 1170–1172. 10.1645/GE-2828.1 21736477

[23] Jithendran KP, Bhat TK. 1996. Prevalence of dicrocoeliosis in sheep and goats in Himachal Pradesh, India. Veterinary Parasitology, 61(3–4), 265–271. 8720564 10.1016/0304-4017(95)00834-9

[24] Liaw A, Wiener M. 2002. Classification and regression by randomForest. R news, 2(3), 18–22.

[25] Manga-González MY, Ferreras MC. 2019. Dicrocoeliidae family: Major species causing veterinary diseases. Adv Exp Med Biol, 1154, 279–319. PMID: 31297766. 31297766 10.1007/978-3-030-18616-6_10

[26] Mateo RG, Felicísimo ÁM, Muñoz J. 2010. Effects of the number of presences on reliability and stability of MARS species distribution models: the importance of regional niche variation and ecological heterogeneity. Journal of Vegetation Science, 21(5), 908–922.

[27] Meshgi B, Majidi-Rad M, Hanafi-Bojd AA, Kazemzadeh A. 2019. Predicting environmental suitability and geographical distribution of *Dicrocoelium dendriticum* at littoral of Caspian Sea: an ecological niche-based modeling. Preventive Veterinary Medicine, 170, 104736. 31421502 10.1016/j.prevetmed.2019.104736

[28] Morgan E, Charlier J, Hendrickx G, Biggeri A, Catalan D, von Samson-Himmelstjerna G, Demeler J, Müller E, van Dijk J, Kenyon F, Skuce P, Höglund J, O’Kiely P, van Ranst B, de Waal T, Rinaldi L, Cringoli G, Hertzberg H, Torgerson P, Wolstenholme A, Vercruysse J. 2013. Global change and helminth infections in grazing ruminants in Europe: impacts, trends and sustainable solutions. Agriculture, 3(3), 484–502.

[29] Musella V, Catelan D, Rinaldi L, Lagazio C, Cringoli G, Biggeri A. 2011. Covariate selection in multivariate spatial analysis of ovine parasitic infection. Preventive Veterinary Medicine, 99(2–4), 69–77. 21167615 10.1016/j.prevetmed.2010.11.012

[30] O’Donnell MS, Ignizio DA. 2012. Bioclimatic predictors for supporting ecological applications in the conterminous United States. U.S. Geological Survey Data Series, 691, 10.

[31] Otranto D, Traversa D. 2003. Dicrocoeliosis of ruminants: a little known fluke disease. Trends in Parasitology, 19(1), 12–15. 12488216 10.1016/s1471-4922(02)00009-0

[32] Phelan P, Morgan ER, Rose H, Grant J, O’Kiely P. 2016. Predictions of future grazing season length for European dairy, beef and sheep farms based on regression with bioclimatic variables. Journal of Agricultural Science, 154(5), 765.

[33] Robinson TP, William Wint GR, Conchedda G, Van Boeckel TP, Ercoli V, Palamara E, Cinardi G, D’Aietti L, Hay SI, Gilbert M. 2014. Mapping the global distribution of livestock. PLoS One, 9(5), e96084. 24875496 10.1371/journal.pone.0096084 PMC4038494

[34] Rojo-Vázquez FA, Meana A, Valcárcel F, Martínez-Valladares M. 2012. Update on trematode infections in sheep. Veterinary Parasitology, 189(1), 15–38. 22521973 10.1016/j.vetpar.2012.03.029

[35] Scala A, Tamponi C, Dessì G, Sedda G, Sanna G, Carta S, Corda A, Jacquiet P, Varcasia A, Ligios C. 2019. Dicrocoeliosis in extensive sheep farms: a survey. Parasites & Vectors, 12(1), 1–7. 31300008 10.1186/s13071-019-3609-2 PMC6625022

[36] Shinggu PA, Olufemi OT, Nwuku JA, Baba-Onoja EBT, Iyawa PD. 2019. Liver flukes egg infection and associated risk factors in white Fulani cattle slaughtered in Wukari, southern Taraba State, Nigeria. Advances in Preventive Medicine, 2019, 5, Article ID 2671620. 10.1155/2019/2671620 PMC6444236 31016046

[37] Stockwell DRB, Peterson AT. 2002. Effects of sample size on accuracy of species distribution models. Ecological Modelling, 148, 1–13.

[38] Syfert MM, Smith MJ, Coomes DA. 2013. The effects of sampling bias and model complexity on the predictive performance of MaxEnt species distribution models. PLoS One, 8(2), e55158. 23457462 10.1371/journal.pone.0055158 PMC3573023

[39] Team R. 2019. R: a language and environment for statistical computing. Vienna, Austria: R Foundation for Statistical Computing.

[40] Walker PA, Cocks KD. 2009. HABITAT: a procedure for modelling a disjoint environmental envelope for a plant or animal species. Global Ecology and Biogeography, 1(4), 108–118.

[41] Wisz MS, Hijmans RJ, Li J, Peterson AT, Graham CH, Guisan A, Group NPSDW. 2008. Effects of sample size on the performance of species distribution models. Diversity and Distributions, 14(5), 763–773.

[42] Zurell D, Franklin J, König C, Bouchet PJ, Dormann CF, Elith J, Fandos G, Feng X, Guillera-Arroita G, Guisan A. 2020. A standard protocol for reporting species distribution models. Ecography, 43(9), 1261–1277.

[43] R Core Team. 2017. R: A Language and Environment for Statistical Computing. https://www.R-project.org/.

